# MFTR-Net: A Multi-Level Features Network with Targeted Regularization for Large-Scale Point Cloud Classification

**DOI:** 10.3390/s23083869

**Published:** 2023-04-10

**Authors:** Ruyu Liu, Zhiyong Zhang, Liting Dai, Guodao Zhang, Bo Sun

**Affiliations:** 1 School of Information Science and Technology, Hangzhou Normal University, Hangzhou 311121, China; 2 Quanzhou Institute of Equipment Manufacturing, Haixi Institutes, Chinese Academy of Sciences, Quanzhou 362000, China; 3 School of Computer Science and Engineering, Tianjin University of Technology, Tianjin 300384, China; 4 Department of Digital Media Technology, Hangzhou Dianzi University, Hangzhou 310018, China

**Keywords:** 3D feature, CNN, TargetDrop, point cloud classification

## Abstract

There are some irregular and disordered noise points in large-scale point clouds, and the accuracy of existing large-scale point cloud classification methods still needs further improvement. This paper proposes a network named MFTR-Net, which considers the local point cloud’s eigenvalue calculation. The eigenvalues of 3D point cloud data and the 2D eigenvalues of projected point clouds on different planes are calculated to express the local feature relationship between adjacent point clouds. A regular point cloud feature image is constructed and inputs into the designed convolutional neural network. The network adds TargetDrop to be more robust. The experimental result shows that our methods can learn more high-dimensional feature information, further improving point cloud classification, and our approach can achieve 98.0% accuracy with the Oakland 3D dataset.

## 1. Introduction

With the continuous development of intelligent driving and remote sensing information technology, it is an important but challenging task to accurately identify objects in large-scale three-dimensional (3D) data. Laser radar technology is a key method to perceive a 3D environment through laser scanning [1], which can be divided into airborne laser radar and vehicle-mounted laser radar. Point cloud data with different structures obtained through different laser radar scanning reflect the geometric structure and spatial distribution of objects in 3D space, but an irregular point cloud makes the feature extraction of the point cloud challenging.

The main goal of a large-scale point cloud classification task is to classify the point cloud data scanned by the radar sensors, and label different objects in the point cloud, such as buildings, roads, vehicles, pedestrians, and vegetation [2]. The traditional method of point cloud classification is mainly based on manual feature extraction, which suffers from low classification accuracy, such as calculating eigenvalues based on adjacent fields of the original point cloud and classifying eigenvalues using Random Forest (SVM) [3] and decision tree (DT) methods [4]. With the development of deep learning (DL), DL can learn task-related features from a large amount of environmental data. More and more scholars introduced DL into point cloud classification tasks. Guo et al. [5] reviewed the latest progress of point-cloud-related tasks based on DL. Sarker et al. [6] used the 3D classifier PointNet to verify robustness by generating adversarial inputs; however, the verifier cannot be extended to more complex 3D vision. Venkanna et al. [7] studied weights of the average of the first two checkpoints of PointNet to improve classification accuracy. Min et al. [8] proposed a masked autoencoding framework Voxel-MAE for pre-training large-scale point clouds. Zhu et al. [9] proposed a global relation-aware attention module (GRA) and spatial relation-aware attention module (SRA) to learn global spatial and channel-wise relationships among spatial positions and feature vectors. Wen et al. [10] explored local graphs in the spectral domain to accelerate point cloud classification tasks. Zhang et al. [11] proposed an effective convolution operator to keep point cloud convolutions invariant to vastly improve feature descriptiveness. Zhang et al. [12] presented an effective point cloud classification method based on the MLP (multilayer perception) network. Feature extraction of the point cloud in this method lacks the consideration of the combination of 3D point cloud feature values and 2D feature values. A pure MLP structure calculation will amplify 2D features, resulting in the loss of part of the feature information.

Based on but different from our previous work [12], we design a point cloud classification framework based on the U-Net network, which can effectively retain feature information in the propagation of the network layer compared with MLP. Our method uses a multi-level features network with targeted regularization, named MFTR-Net, which can effectively accelerate the stability of the results and achieve a better classification effect. The main contributions of this paper are as follows:

(1) We propose a new feature construction method for large-scale point clouds, which can effectively calculate the multi-level local feature information of the point cloud from the irregular point cloud data.
(2) We present the MFTR-Net framework for point cloud classification. The designed encoder–decoder model can effectively extract the local feature information of the point cloud from the input feature map, and strengthen the attention to spatial information.
(3) We conduct extensive experiments on the 3D point cloud dataset, Oakland. The experimental results show that the proposed MFTR-Net has achieved satisfactory results in large-scale point cloud classification tasks.

## 2. Related Work

Traditional machine learning methods for large-scale point cloud classification are carried out by screening appropriate features of a large-scale point cloud, such as computational geometry, spectrum, and texture. Li et al. [13] proposed a dynamic feature aggregation (DFA) method that can transfer information by constructing local graphs in the feature domain without spatial constraints. Venkanna et al. [7] extracted point cloud geometric features, plane features, and intensity features combined with Random Forest (RF) and Conditional Random Field (CRF) to optimize classification results. With the explosive growth of 3D data, traditional methods cannot extract more effective information from a large number of point cloud data.

Deep learning technology relies on convolution neural networks (CNN) to effectively learn task-related features from a large amount of data and plays an important role in both 2D image and 3D point cloud tasks, so it is also applied to point cloud classification tasks. Chen et al. [14] proposed an unsupervised deep neural architecture, Flattening Net, which converts different point clouds into color images, and then classifies different color images to achieve the classification of variable point clouds. This method can effectively reduce network parameters and achieve better classification results. Melnyk et al. [15] proposed a learnable descriptor for rotation and reflection invariant 3D point cloud classification. This method converts 3D point cloud data into a 4D data representation to perform a dimension upgrading operation, which effectively overcomes the critical problem of rotation invariance of point cloud classification tasks. The subsequent network then extracts the rotation invariance feature information of the point cloud to achieve good results. Zhao et al. [16] designed a highly expressed point converter layer for point cloud classification, which proves that the converter model can also achieve excellent results in point cloud classification tasks. Wang et al. [17] proposed a semi-supervised cross-domain learning method. By sampling the rotating images of any point cloud from multiple views, these images are regarded as enhancement modules in point cloud classification, which makes up for the insufficient extraction of some occlusion feature information in point cloud classification. Wong et al. [18] presented an end-to-end encoder–decoder network named GACNN to capture multiscale features of point clouds and therefore realize more accurate point cloud classification. Park et al. [19] proposed a fast voxel-based semantic segmentation model using point convolution and 3D sparse convolution. After feature extraction in a point cloud classification task, it can accelerate feature propagation. Yang et al. [20] proposed a supervised contrastive point cloud classification method to implement embedding feature distribution refinement by improving intra-class compactness and inter-class separability, which solves the confusion problem caused by slight inter-class variations and the confusion problem caused by small inter-class compactness and inter-class separability. Zhang et al. [12] proposed a method that automatically learns a data augmentation strategy using bilevel optimization, minimizing a base model’s loss on a validation set when the augmented input is used for training the model. This can reduce overfitting and improve learning performance.

The above point cloud classification methods mainly extract features directly from irregular and disordered point clouds. On the one hand, it is difficult to learn the regular 3D features. On the other hand, it will also ignore local characteristics from the plane view, resulting in incomplete point cloud feature information. In contrast, the proposed MFTR-Net extracts point cloud features from both 3D and 2D perspectives and thus effectively improves the accuracy of large-scale point cloud classification. In addition, the networks in the above related work treat redundant point cloud data indiscriminately, while our method introduces an attention mechanism to make the network pay more attention to the key features in the point cloud information, thus improving the accuracy of the point cloud classification task.

## 3. MFTR-Net: A Multi-Level Features Network with Targeted Regularization for Large-Scale Point Cloud Classification

### 3.1. Feature Construction for Point Clouds

**Figure 1:**
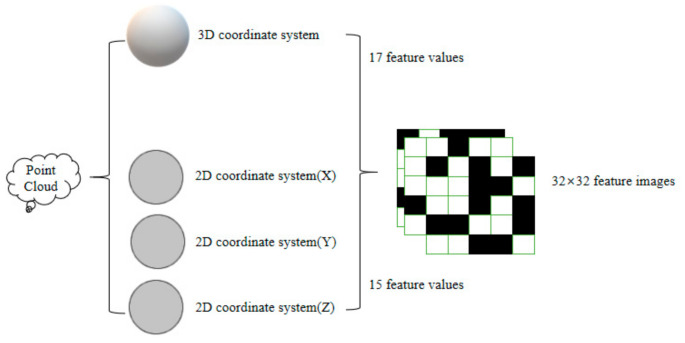
Generation of point cloud feature images.

Large-scale point clouds are generally irregular and disordered, which will seriously affect the final result of point cloud classification. Therefore, we consider that transforming irregular point cloud data into regular image data may overcome this problem, so we design a point cloud feature construction method based on a multi-level feature combination. This method calculates the eigenvalues of the adjacent points of an unordered 3D point cloud and then combines the obtained eigenvalues into one ordered 2D point cloud feature image to represent the point cloud features as richly as possible. Specifically, as shown in Figure 1, this method calculates the local eigenvalues of 100 adjacent points around each point through the Kdtree method to obtain 3D eigenvalues in 3D space and 2D eigenvalues on three different coordinate axes. The 3D eigenvalues vector and the 2D eigenvalues vector projected in three different coordinate systems are combined to form a point cloud feature matrix, and the obtained point cloud feature matrix is represented as current point cloud feature information.

The point cloud feature image is visualized by a feature matrix, which is composed of the obtained 3D and 2D point cloud feature values, as shown in Table 1. We arrange the obtained 3D and 2D eigenvalues horizontally and vertically into a 32 × 32 size feature matrix, which is a positive definite matrix. We normalize the values in the feature matrix to (0, 1), so that we can screen out some outlier data and normalize them to (0–255), and form white and black point cloud feature images. Subsequently, the obtained point cloud feature image is input into the designed neural network for classification, and the network finally outputs the various categories of the point cloud, thus realizing point cloud classification.

We set *P* as a point (*x*, *y*, *z*) in 3D space, and $\;\lambda_{1}$, $\lambda_{2}$, and $\lambda_{3}$, which represent the distribution pattern of the point cloud, namely divergent, areal, and linear distribution, are calculated as follows.

(1) $$\lambda_{1}=\left[\begin{array}{ccc} x_{1} & y_{1} & z_{1}\\ x_{2} & y_{1} & z_{1}\\ \begin{array}{c} \vdots \\ x_{n} \end{array} & \begin{array}{c} \vdots \\ y_{n} \end{array} & \begin{array}{c} \vdots \\ z_{n} \end{array} \end{array}\right]$$

(2) $$\lambda_{2}=\left[\begin{array}{ccc} \;x_{1}^{\prime } & y_{1}^{\prime } & z_{1}^{\prime }\\ x_{2}^{\prime } & y_{2}^{\prime } & z_{2}^{\prime }\\ \begin{array}{c} \vdots \\ x_{n}^{\prime } \end{array} & \begin{array}{c} \vdots \\ y_{n}^{\prime } \end{array} & \begin{array}{c} \vdots \\ z_{n}^{\prime } \end{array} \end{array}\right]$$

(3) $$\lambda_{3}=\frac{1}{n}{\left(\lambda_{1}-\lambda_{2}\right)}^{T}\left(\lambda_{1}-\lambda_{2}\right)$$

(4) $$M_{n}\left(dist\left(P_{n}-P_{n}^{\prime }\right)\right),n\in x,y,z$$

(5) $$N_{n}\left(dist\left(P_{n}-P_{n}^{\prime }\right)\right),n\in x,y,z$$

$M_{x}\;,\;M_{y}\;,\;M_{z}$ represent the adjacent max radius of *P* on the x, y, and z axes;$\;N_{x}\;,\;N_{y}\;,\;N_{z}$ represent the adjacent min radius of *P* on the x, y, and z axes; *P* represents the current point; and $P^{\prime }$ represents the next point.

In order to effectively express the feature information of the current point cloud, we select some feature information that can effectively represent the differences between different point cloud objects, such as change of curvature value $C_{\lambda }$, linearity value $L_{\lambda }$, planarity value $P_{\lambda }$, and scattering value $S_{\lambda }$.

(6) $$C_{\lambda }=\frac{\lambda_{3}}{\lambda_{1}+\lambda_{2}+\lambda_{3}}$$

(7) $$L_{\lambda }=\frac{\lambda_{1}-\lambda_{2}}{\lambda_{1}}$$

(8) $$P_{\lambda }=\frac{\lambda_{2}-\lambda_{3}}{\lambda_{1}}$$

(9) $$S_{\lambda }=\frac{\lambda_{3}}{\lambda_{1}}$$

In the 3D coordinate system, the large-scale point cloud will contain some other local feature information, such as the Omnivariance value of local 3D shape $O_{\lambda }$, Eigenentropy value $E_{\lambda }$, Anisotropy $A_{\lambda }$, Eigenentropy $E_{\lambda }$, Eigenentropy of eigenvalues $T_{\lambda }$, Verticality value D, Variance of z V, and EVs_3D value Q.

(10) $$O_{\lambda }=\sqrt[{3}]{e_{1}e_{2}e_{3}}$$

(11) $$A_{\lambda }=\frac{e_{1}-e_{3}}{e_{1}}$$

(12) $$E_{\lambda }=-\sum_{i=1}^{3}e_{i}ln\left(e_{i}\right)$$

(13) $$T_{\lambda }=\frac{2}{\pi }arctan\left(\lambda_{1}+\lambda_{2}+\lambda_{3}\right)\;$$

(14) $$D=\frac{k+1}{\pi \cdot maxradias_{k}^{2}},$$

(15) $$V=1-\left|n_{z}\right|,$$

(16) Q=Eig(P(x,y,z)) 

In order to extract more effective eigenvalues, we calculated five eigenvalues for point clouds projected on three different coordinate axes on the 2D plane. The 2D feature value is calculated based on the x and y values of the adaptive adjacent point cloud, regardless of the z value of the point cloud and the eigenvalues in the z direction.

(17) $$r_{k}=\sqrt{{\left(x-x_{k}\right)}^{2}+{\left(y-y_{k}\right)}^{2}}$$

(18) $$D_{2}=\frac{k+1}{\pi r_{k}^{2}}$$

(19) $$R_{\lambda ,2D}=\frac{\lambda_{2,2D}}{\lambda_{1,2D}}$$

(20) $$Evratio=E_{\lambda 2D}\left(axisa\right)/E_{\lambda 2D}\left(axisb\right)$$

(21) $$S_{2}=\lambda_{1,1D}+\lambda_{2,2D}$$

where $r_{k}\;$ represents 2D circle neighborhood radius, $D_{2}\;$ is 2D local point density, and $R_{\lambda ,2D}$ represents the ratio of the 2D covariance matrix. The Evratio value represents the eigenvalue entropy ratio of the coordinate system, and $S_{2}$ represents the sum of the feature values.

### 3.2. TargetDrop-Based MFTR-Net

**Figure 2:**
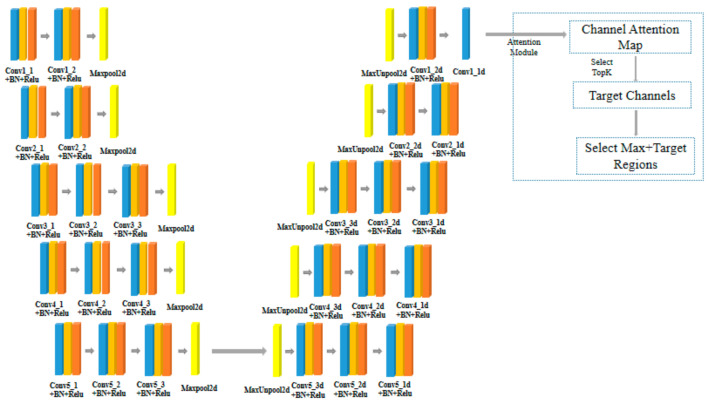
MFTR-Net framework.

MFTR-Net is based on the U-Net framework with the attention mechanism of TargetDrop [21]. MFTR-Net is an encoder and decoder structure and consists of a downsampling network and a corresponding upsampling network, as shown in Figure 2. First, the convolution layer downsamples the input point cloud feature images. Then the downsampling network is gradually deepened to extract the features layer by layer. The downsampling network includes 13 convolution layers. Each downsampling network layer has a corresponding upsampling network layer, so the upsampling network also has 13 convolution layers. Then, the features of each layer are inversely used for upsampling. The upsampled output is sent to the TargetDrop attention module. TargetDrop mainly processes features to increase the proportion of high-dimensional features. Reasonable weight distribution for some high-dimensional features can improve the extraction ability of the network for high-dimensional feature information. Finally, the class probability distribution results of each pixel are independently generated through the entire connection layer.

**Figure 3:**
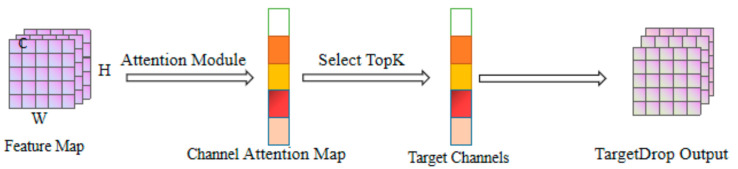
The pipeline of TargetDrop.

In MFTR-Net, each convolution layer has a filter to extract the feature information of the image, and combine the obtained features into a group of feature information maps. These feature maps are further normalized to (0, 1) and propagated in the network. Then, the Relu function is used to discard part of the information of the feature maps and reduce over-fitting of the network layer. The window performs maximum pooling, and the output results are subject to secondary downsampling. Subsampling generates significant input image context (spatial window) for each pixel in the feature map. Its input feature map uses the maximum pool index stored from the corresponding encoder feature map. Multiple Max pooling and downsampling layers can reduce the calculation of parameter amount and achieve translation invariance; however, they suffer from the loss of feature information (Figure 3).

After inputting the feature images into the TargetDrop module, we obtain a channel attention map through the attention layer. Then, TopK features in the channel attention map are used to select the high-dimensional feature information, and give this high-dimensional feature a higher weight ratio to obtain the mask we need.

In the final calculation of the network backpropagation loss function (Formula (22)), *Z* represents the value of comparison loss in the propagation process of the network layer, and *y* represents the value of classification loss. When the point cloud characteristic value is transferred to the layer, it will be divided into the calculation of the contrast loss value [22]. Each adjacent node around the current node is taken as a positive sample, and the remaining nodes are taken as negative samples. Through this method, the effective positive sample can be closer to the target node, thus completing the whole attention mechanism, focusing on those features that contain important information, in which the adjacent contrast loss value of the *i*th node can be expressed as:

(22) $$l_{i}=-log\frac{\sum_{j=1}^{B}1_{j\neq i}\gamma_{ij}exp\left(sim\left(z_{i},z_{y}\right)/\tau \right)}{\sum_{k=1}^{B}1_{k\neq i}exp\left(sim\left(z_{i},z_{k}\right)/\tau \right)}$$

The loss function of MFTR-Net consists of adjacent contrastive loss (NC) and Cross-Entropy (CE) loss, and *α* is the weighting coefficient to balance $loss_{NC}$ and $loss_{CE}$.

(23) $$loss_{NC}=\alpha \frac{1}{B}\overset{B}{\underset{i=1}{\sum }}l_{i}$$

(24) $$loss_{final}=loss_{CE}+loss_{NC}$$

### 3.3. MFTR-Net for Large-Scale Point Cloud Classification

**Figure 4:**
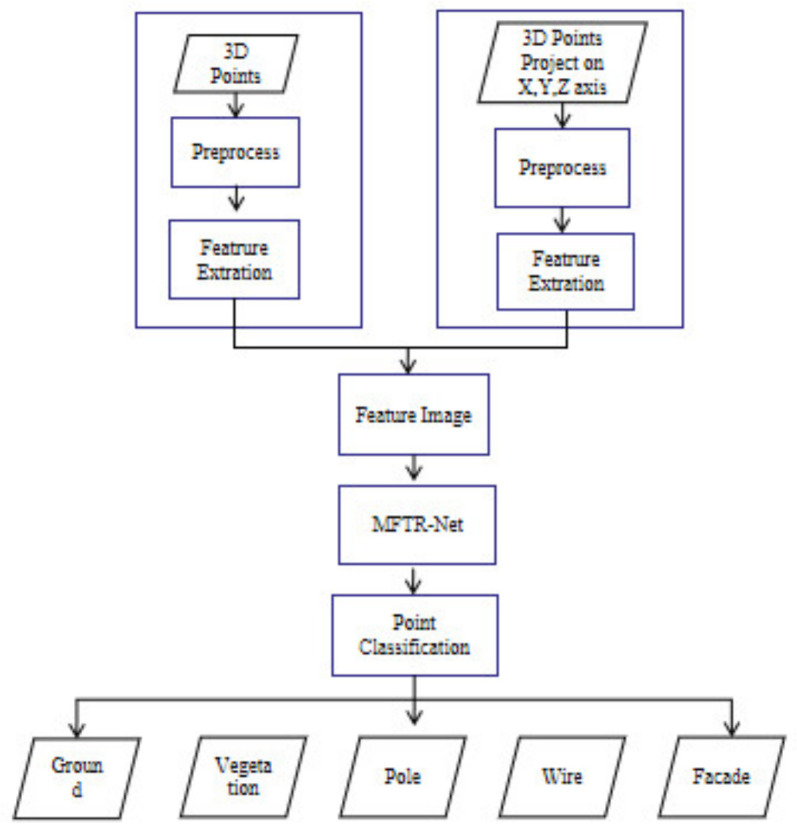
Flow chart of point cloud classification using MFTR-Net.

The complete process and overall flow of large-scale point cloud classification using the proposed MFTR-Net are shown in Figure 4. First, the flow chart is divided into two branches. One is the 3D features branch, which calculates the corresponding eigenvalues of the current point cloud in the 3D coordinate system, and the other is the 2D features branch, which calculates the point cloud eigenvalues projected on the 2D coordinate system on three different planes. Then the total of 32 eigenvalues are constructed into point cloud feature images (32 × 32). The obtained point cloud feature images are input into the MFTR-Net classification network. The network calculates the probability of the classification result of each category and outputs the final corresponding category with classification accuracy.

**Figure 5:**
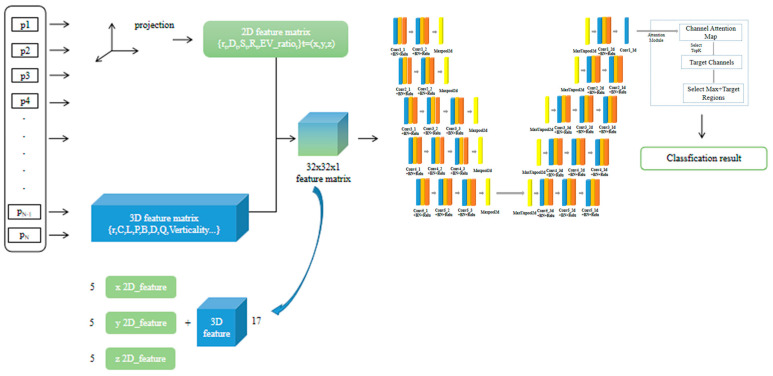
MFTR-Net for large-scale point cloud classification.

In the feature construction part, a dimension reduction method is adopted for processing large-scale point clouds. The original 3D point cloud data [batchsize, num points, 3] are converted into 2D point cloud feature image data [batchsize, height, width, 1]. Then the 2D point cloud feature image is input into MFTR-Net (Figure 5). During the processing, features are extracted through the convolution layer of the encoder module, and the receptive field is increased through pooling. Then, deconvolution in the decoder module enables the feature map to be reproduced and restored to the original size of the image. Finally, the weight of the extracted convolution features is allocated through the TargetDrop method so that the whole network pays more attention to some high-dimensional feature information, thus ensuring the point cloud classification’s overall accuracy.

## 4. Analysis of Experimental Results

In this work, we implement a proposed network in the Tensorflow framework. All the training and testing platforms are Ubuntu 18.04 with Intel i7-4790 and NVIDIA RTX 2070, under Python 3.7, CUDA 10.0, CUDNN7.6.4, Pytorch 0.6, and 256GB of mainframe running memory. We train our model for 120 epochs, and the batch size is 100. The learning rate starts at 0.001 and decays at 0.7 per 50k iterations. A series of comparison experiments are carried out on the 3D point cloud dataset, Oakland [23], to verify the effectiveness and robustness of the MFTR-Net network. The Oakland dataset includes five labels: Vegetation, Pole, Facade, Ground, and Wire. The number of samples on each label is shown in Table 2.

**Figure 6:**
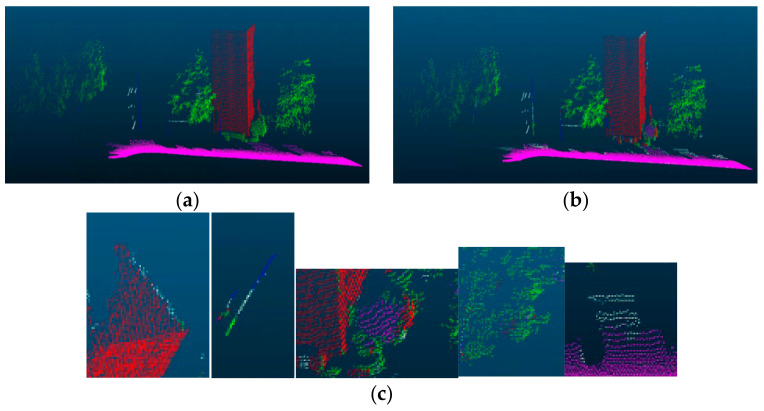
Visualization of the classification result. (**a**) Test dataset ground truth, (**b**) Classification result of our method, (**c**) Details of our classification result.

We compared accuracy indicators with SoTA methods [24,25,26,27,28,29] on the Oakland dataset. These methods [24,25] are processed by directly inputting the point cloud into the network framework. References [25,26] convert the point cloud into binary feature images; however, the subsequent network framework needs to be further improved. Our method is superior to other methods in the classification accuracy of building categories and overall datasets, despite some results not achieving the best in some categories. As shown in Table 3, the bold numbers indicate the best effect for the current category. Our method achieves 98.0% accuracy in the Oakland datasets. The visualization results of certain categories are shown in Figure 6: green represents plants, blue represents wires, white represents poles, purple represents the ground, and red represents buildings.

From the comparison data in Table 3 and the results shown in Figure 6, the lowest classification accuracies of the two categories of Pole and Wire in the whole category are 21.5% and 20.1%, respectively, which is lower than other methods and cannot achieve a good result. The main reason for this phenomenon is that the percentage of these points in the whole dataset is relatively small. Furthermore, after projection from different views, the data overlap, which makes it difficult to calculate the eigenvalues of the current category based on the surrounding point cloud. However, for some cases with a large number of categories, our method can effectively extract the feature information of the current category. The classification accuracy of our building and ground categories is relatively high and can achieve a competitive effect. Moreover, our overall effect is also better than other methods, and the overall classification accuracy is stable at about 98.0%.

**Figure 7:**
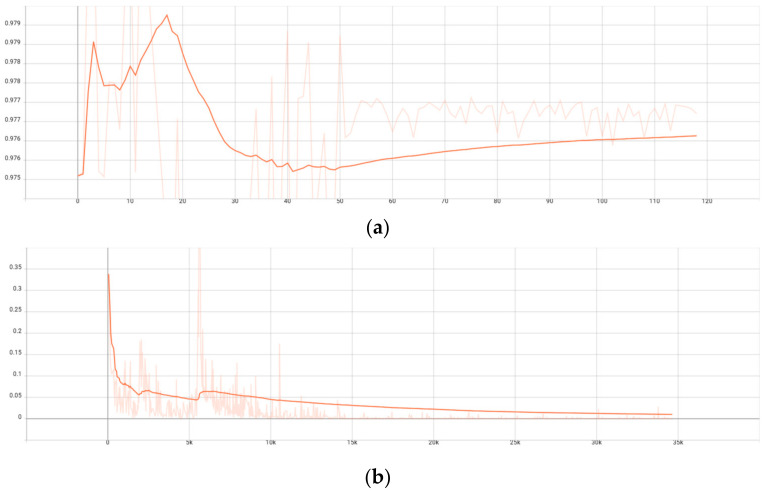
Classification result of MFTR-Net. (**a**) Accuracy flow chart of MFTR-Net, (**b**) Loss flow chart of MFTR-Net.

Figure 7 is the broken-line chart of our network framework on the tensorboard. From the chart, we can see that the accuracy of our network framework has gradually stabilized from epoch = 40 and gradually improved with the learning rate.

## 5. Ablation Study

**Figure 8:**
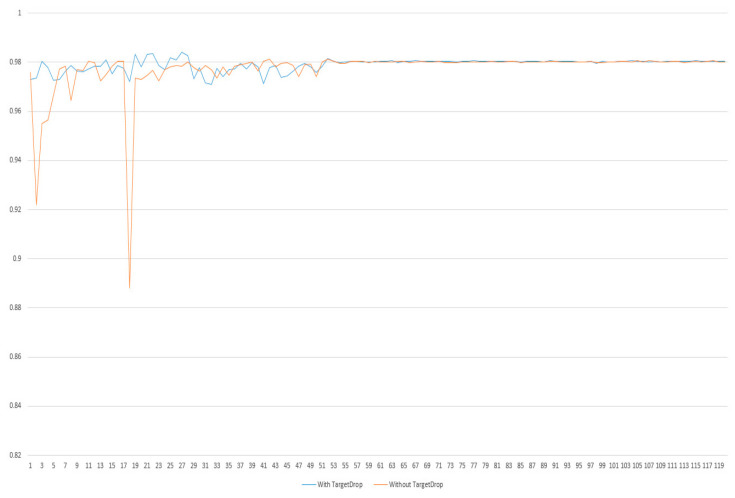
Ablation experiment results of TargetDrop.

In order to evaluate the impact of the current TargetDrop on the result of the overall point clouds classification network, we design ablation experiments to compare the network with and without the TargetDrop module (Figure 8). From the accuracy results, we can see that the effect of the attention mechanism module without TargetDrop fluctuates greatly and does not form a stable trend in the early stage. From the whole comparison effect, we can see that TargetDrop is necessary, which can accelerate the stability of the entire network framework.

The results in Table 4 show that residual learning is more effective than the warm-up training strategy in our framework, a check mark indicates that the method is currently used.Therefore, we equip deep learning for point cloud classification, which makes the proposed method easily optimized without extra training strategies.

In the experiment of feature construction, we design eight different comparative experiments by calculating the point cloud features in the 3D coordinate system and in three 2D projection plane coordinate systems: (1) point cloud classification results only using the 3D point cloud features; (2) point cloud classification results using 2D point cloud eigenvalues and 3D point cloud eigenvalues projected along the *x*-axis direction; (3) point cloud classification using 2D point cloud features and 3D point cloud features projected along the *y*-axis direction; (4) point cloud classification using 2D point cloud features and 3D point cloud features projected along the *z*-axis direction; (5) point cloud classification using 3D point cloud features and all 2D point cloud features; (6) point cloud classification without using 2D point cloud features projected along the *x*-axis direction and 3D point cloud features; (7) point cloud classification results without using the 2D point cloud features projected along the *y*-axis direction and the 3D point cloud features; and (8) point cloud classification results without using 2D point cloud features projected along the *z*-axis direction and 3D point cloud features. Other comparison groups are shown in Table 5, the bold numbers indicate the best effect for the current category. 

**Figure 9:**
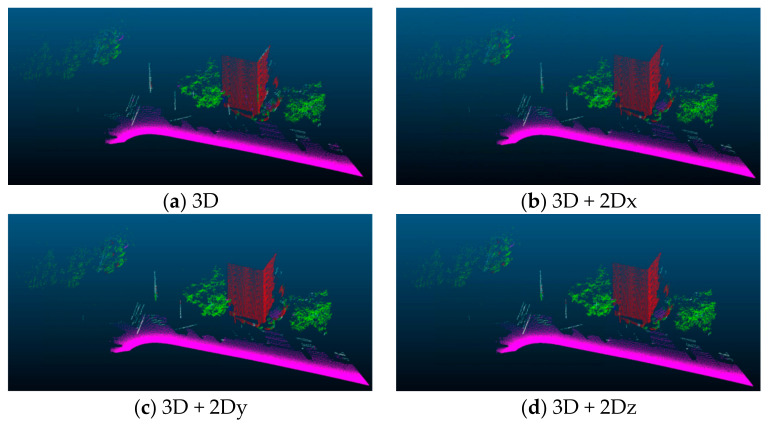

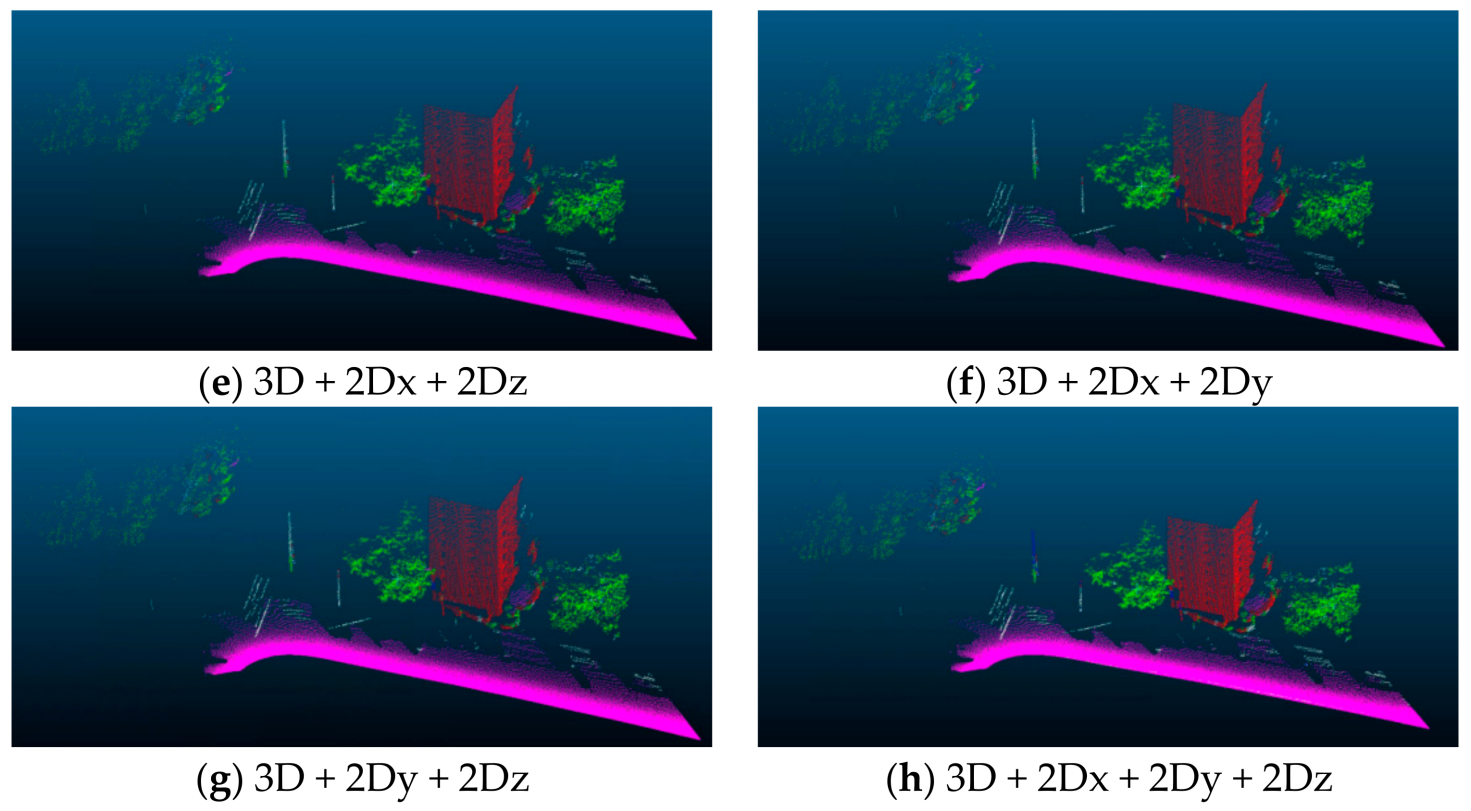
Visualization result of the comparison groups.

Figure 9 shows the visualization results of different groups of experiments. We can see from the results that these groups of experiments mainly produce different visualization results around three categories: poles, wires, and plants. We can see from the figure that there is a large degree of classification error between wires and poles. Some of the wires are surrounded by trees, so there is a partial classification error. This leads to low classification accuracy of wires and poles.

## 6. Conclusions

The MFTR-Net network framework model can effectively improve classification accuracy for large-scale point clouds. We combine 3D and 2D features to effectively retain point cloud feature information and introduce the attention mechanism TargetDrop to further enhance the point cloud feature information so as to realize accurate classification for large-scale point clouds. However, the proposed method still has some room for improvement. In the feature construction of the point cloud, it is difficult to ensure that the calculated point cloud eigenvalues can fully express the point cloud feature information, resulting in the point cloud feature image being affected by the point cloud’s disorder and rotation invariance. In addition, the real-time nature of point cloud classification determines whether it can be deployed on hardware platforms. In the future, we will improve the speed of point cloud classification so that our current effects can run in real-time on hardware.

## Figures and Tables

**Table 1:** Point cloud feature values.

Type	Components
3D eigenvalues	$\;M_{x}\;,\;M_{y}\;,\;M_{z}\;,\;\;N_{x},\;N_{y},\;N_{z},\;C_{\lambda },\;L_{\lambda },\;P_{\lambda }\;,\;S_{\lambda }$ $O_{\lambda },\;A_{\lambda }\;,\;E_{\lambda }\;,\;T_{\lambda },\;D\;,\;Q\;,\;V$
2D eigenvalues	$r_{k}\;,\;D_{2}\;,\;R_{\lambda \;,\;2D}\;,\;Evratio\;,\;S_{2}$

**Table 2:** Oakland dataset.

Label	Training Dataset	Test Dataset
Vegetation	14,441	9278
Wire	2571	481
Pole	1086	368
Ground	4713	71,863
Facade	14,121	7821
Total	36,932	89,811

**Table 3:** Comparison accuracy of Oakland dataset (%).

	Pole	Vegetation	Wire	Ground	Facade	OA
Cabo [24]	**77.3**	80.6	80.4	99.2	92.9	86.1
Chen-Chieh [25]	-	-	-	**100.0**	94.7	97.0
Wang [26]	68.4	80.6	92.9	98.3	71.1	94.7
Wang [27]	70.1	80.5	**93.0**	98.2	70.9	94.6
Ekaterina [28]	28.7	**97.4**	12.5	98.2	90.8	91.6
Kumar [29]	70.9	94.7	-	97.9	94.4	-
Our method	21.5	93.8	20.1	99.5	**9** **8.** **1**	**9** **8.** **0**

**Table 4:** Warm-up strategy in our framework.

Warm-Up	Deep learning	Accuracy
√	-	89.5
√	√	98.3
-	-	88.1
-	√	98.0

**Table 5:** Multi-level features accuracy of Oakland dataset (%).

	Pole	Vegetation	Wire	Ground	Facade	OA
3D	0.0	84.1	**30.3**	**99.** **7**	92.4	96.7
3D + 2Dx	10.0	97.4	8.2	94.4	82.0	85.6
3D + 2Dy	0.0	87.6	0.0	65.3	0.0	61.3
3D + 2Dz	0.0	99.7	0.0	99.4	0.0	89.8
3D + 2Dx + 2Dz	0.0	**99.9**	0.0	99.4	0.0	89.8
3D + 2Dx + 2Dy	**25.0**	**99.9**	0.0	97.1	38.0	88.5
3D + 2Dy + 2Dz	16.8	**99.9**	0.0	99.2	0.0	89.7
3D + 2Dx + 2Dy + 2Dz	21.5	93.8	20.1	99.5	**9** **8.** **1**	**9** **8.** **0**

## Data Availability

Not applicable.
